# Diagnostic performance of chest radiography in high COVID-19 prevalence setting: experience from a European reference hospital

**DOI:** 10.1007/s10140-021-01946-x

**Published:** 2021-07-03

**Authors:** Nicola Flor, Lorenzo Saggiante, Anna Paola Savoldi, Renato Vitale, Giovanni Casazza, Paolo Villa, Anna Maria Brambilla

**Affiliations:** 1 U.O. di Radiodiagnostica, Ospedale L. Sacco ASST Fatebenefratelli Sacco, Via Giovanni Battista Grassi, 74, 20157 Milan, MI Italy; 2 Unità Operativa di Radiologia, ASST Fatebenefratelli Sacco, Luigi Sacco University Hospital, Via Giovanni Battista Grassi, 74, 20157 Milan, Italy; 3 Postgraduation School in Radiodiagnostics, Università degli Studi di Milano, Via Festa del Perdono, 7, 20122 Milan, MI Italy; 4 Dipartimento di Scienze Biomediche e Cliniche “L. Sacco”, Università degli Studi di, Milan, MI Italy; 5 U.O. di Medicina e Chirurgia d’Accettazione e d’Urgenza, Ospedale L. Sacco ASST Fatebenefratelli Sacco, Via Giovanni Battista Grassi, 74, 20157 Milan, MI Italy

**Keywords:** Radiology/radiography, Pneumonia

## Abstract

**Purpose:**

The study’s aim is to analyse the diagnostic performance of chest radiography (CXR) in patients with suspected coronavirus disease 19 (COVID-19).

**Methods:**

We retrospectively considered 826 consecutive patients with suspected COVID-19 presenting to our emergency department (ED) from February 21 to March 31, 2020, in a high disease prevalence setting. We enrolled patients who underwent CXR and rhino-oropharyngeal swab for real-time reverse transcription-polymerase chain reaction (rRT-PCR). CXRs were evaluated by an expert radiologist; a second independent analysis was performed by two residents in consensus. All readers, blinded to rRT-PCR results, classified CXRs positive/negative depending on presence/absence of typical findings of COVID-19, using rRT-PCR as reference standard.

**Results:**

We finally analysed 680 patients (median age 58); 547 (80%) tested positive for COVID-19. The diagnostic performance of CXR, interpreted by the expert reader, was as follows: sensitivity (79.0%; 95% CI: 75.3–82.3), specificity (81.2%; 95% CI: 73.5–87.5), PPV (94.5%;95% CI: 92.0–96.4), NPV (48.4%; 95% CI: 41.7–55.2), and accuracy (79.3%; 95% CI: 76.0–82.2). For the residents: sensitivity (75.1%; 95% CI: 71.2–78.7), specificity (57.9%; 95% CI: 49.9–66.4), PPV (88.0%; 95% CI: 84.7–90.8), NPV (36.2%; 95% CI: 29.7–43.0), and accuracy (71.6%; 95% CI: 68.1–75.0). We found a significant difference between the reporting sensitivity (*p* = 0.013) and specificity (*p* < 0.0001) of expert radiologist vs residents. CXR sensitivity was higher in patients with symptom onset > 5 days before ED presentation compared to ≤ 5 days (84.4% vs 70.7%).

**Conclusions:**

CXR showed a sensitivity of 79% and a specificity of 81% in diagnosing viral pneumonia in symptomatic patients with clinical suspicion of COVID-19. Further studies in lower prevalence settings are needed.

**Supplementary Information:**

The online version contains supplementary material available at 10.1007/s10140-021-01946-x.

## Introduction

In the year 2020, the world has seen a steady increase in the number of coronavirus disease 2019 (COVID-19) cases, an infectious disease caused by the recently discovered respiratory pathogen severe acute respiratory syndrome coronavirus 2 (SARS-CoV-2), with over 107 million confirmed cases and over 2.3 million deaths as of February 12, 2021 [1].

Because of the high number of cases, especially during peaks of incidence, managing the emergency has proved challenging in many countries.

Adequate management of the emergency, especially in a setting of high disease prevalence, requires early diagnosis in order to control the spread of the infection, isolating infected patients (either at home or in hospital), and to avoid ED congestion*.*

The reference standard test for the diagnosis of SARS-CoV-2 infection is real-time reverse transcription-polymerase chain reaction (rRT-PCR) on rhino-oropharyngeal swab samples; however, the laboratory procedure is time-consuming and may become a rate-limiting step if there is an increase in demand; moreover, it has a moderate sensitivity, ranging from 60 to 70% [2, 3].

In this context, chest imaging has played an important role in the diagnostic work-up of patients with suspected COVID-19, in association with clinical and laboratory data; it has been particularly helpful in settings where rRT- PCR results were not readily available or in case of discrepancies between negative rRT-PCR results and clinical data [4].

Most radiological papers published since the beginning of this pandemic have focused on chest CT, which has shown the highest sensitivity among medical imaging modalities, despite a low specificity [2, 5–7]. However, concerns have emerged about contamination risks, the need for dedicated transit routes and machines, high radiation exposure, and costs. European and US imaging societies have issued statements advising against a routine use of CT scan as a screening tool [8, 9]; at ED, chest radiography (CXR) and lung ultrasound (LUS) [10] represent alternative imaging modalities with significant advantages, such as lower radiation doses, lower risk of contamination, lower costs, and more widespread availability.

Even though the reported sensitivity of CXR and LUS in the diagnosis of COVID-19 appears to be lower than that of chest CT, the evidence in literature is still poor, as only a limited number of studies focusing on their diagnostic performance have been published [11–14].

Thanks to the experience of our centre, one of the reference hospitals for COVID-19 in [Ospedale Luigi Sacco, Milan, Italy], in which the diagnostic pathway involves the routine use of CXR, reserving chest CT for selected cases, we were able to collect a large consecutive series of CXRs performed in patients with clinical suspicion of COVID-19.

Therefore, the aim of our study is to analyse the diagnostic performance of CXR in the diagnosis of COVID-19, in a large high prevalence cohort.

## Materials and methods

### Study setting and design

This single-centre, retrospective, diagnostic accuracy study was undertaken at the ED of [Ospedale Luigi Sacco, Milan, Italy]. The hospital is a tertiary care centre for infectious diseases, serving a population of 350,000, funded by the government, and free to patients at point-of-care. The city has a strong primary healthcare system, and the hospital has 31 intensive care beds. The ED treats 50,000 patients per annum, median age 65, with an admission rate in the pre-COVID-19 era of 16%.

This study involving human participants was in accordance with the ethical standards of the institutional and national research committee and with the 1964 Helsinki Declaration and its later amendments or comparable ethical standards. The Human Investigation Committee (IRB) of [Ospedale Luigi Sacco, Milan, Italy] approved this study.

The study received no specific grant from funding agencies in the public, commercial, or non-profit sector. Due to the retrospective nature of this study, specific informed consent was waived.

Our study was conducted and reported according to the Standards for Reporting Diagnostic Accuracy (STARD) [15].

### Study population

We considered eligible for inclusion a consecutive series of patients who presented to the ED of our hospital between February 21 and March 31, 2020, with clinical and epidemiological data raising suspicion of COVID-19. At that time, being our hospital a reference centre for COVID-19, almost all ED patients were suspected of having COVID-19, with the exception of a minority of patients who self-presented to the ED with other emergency diseases.

We only included adult patients who underwent both a baseline CXR and a rhino-oropharyngeal swab for rRT- PCR testing within 24 h from admission, being identified as COVID-19 or non-COVID-19 patients in the ED or in the first days of hospitalization. Three investigators collected clinical data (symptoms, comorbidities) and rRT-PCR results from the digital archive of our ED.

### Image analysis

For all patients, only the first CXR acquired at ED admission was evaluated.

All CXRs were acquired as digital radiograms in the isolation wards of our ED with the same portable X-ray unit (Adora, Canon Medical Systems), in two projections (postero-anterior and latero-lateral) when compatible with patients’ conditions, and in one projection (anteroposterior) in seriously ill patients. All the images were stored in a picture archiving and communication system (IMPAX, Agfa Healthcare).

All CXRs were retrospectively and independently evaluated first by a radiologist with more than 20 years of experience. A second analysis was conducted by two radiology residents with 2 years of experience; after an independent evaluation, discussion and consensus resolved any disagreement. All readers were blinded to the rRT-PCR results, while being provided with clinical information (symptoms, respiratory function, comorbidities).

According to the literature [11, 16–18], the main features we considered as suggestive for COVID-19 were interstitial reticular pattern, ground-glass opacities, and extensive consolidations, mostly involving lower sites of both lungs, with a preferred peripheral subpleural distribution. Therefore, readers classified CXRs as positive for COVID-19 if at least one of these alterations was observed.

In order to evaluate the extension and distribution of the disease, each lung was virtually divided into six areas (upper external, upper internal, middle external, middle internal, lower external, lower internal), for a total of 12. Each reader used a worksheet to check for the pattern categories (interstitial reticular pattern, ground-glass opacities and extensive consolidations) in each lung zone.

### Real-time reverse transcription-polymerase chain reaction

For SARS-CoV-2 infection diagnosis, rRT-PCR on rhino-oropharyngeal swabs was used (ELITe InGenius® system and the GeneFinder COVID-19 Plus RealAmp Kit assay; ELITechGroup, France).

For patients who underwent multiple rRT-PCR tests, we considered as confirmed COVID-19 patients those with at least one positive result within 8 days from admission to the ED.

In case of discrepancies between rRT-PCR on one side and clinical data and CXR on the other, clinicians ordered more samples for rRT-PCR testing and serologic tests (IgG); moreover, in few selected cases, chest CT was performed.

### Statistical analysis

Categorical variables were reported as counts and percentages. Continuous variables were represented as mean and standard deviation (SD) or median and interquartile range (IQR), as appropriate. The difference in age between different groups was assessed using Mann-Whitney test, the difference in sex using chi-square test.

Diagnostic accuracy measures (sensitivity; specificity; positive predictive value, PPV; negative predictive value, NPV; positive likelihood ratio, LR+; negative likelihood ratio, LR-; overall accuracy) with corresponding 95% confidence intervals (CI) were calculated using rRT-PCR results as reference standard.

A statistical comparison of sensitivity and specificity of the expert radiologist vs residents was done using McNemar’s test.

For all further analysis, only the CXR interpretation by the expert radiologist was considered.

A comparison of sensitivity and specificity between subgroups (divided according to time from symptoms onset, hospital admission and presence of comorbidities) was performed using Fisher exact test.

Finally, a multivariate logistic regression analysis was performed to assess the contribution of the different CXR areas in identifying COVID-19 patients.

*P* values ≤ 0.05, two sided, were considered statistically significant. All the analyses were performed using SAS statistical software (release 9.4).

## Results

### Patients characteristics and rRT-PCR results

**Fig. 1:**
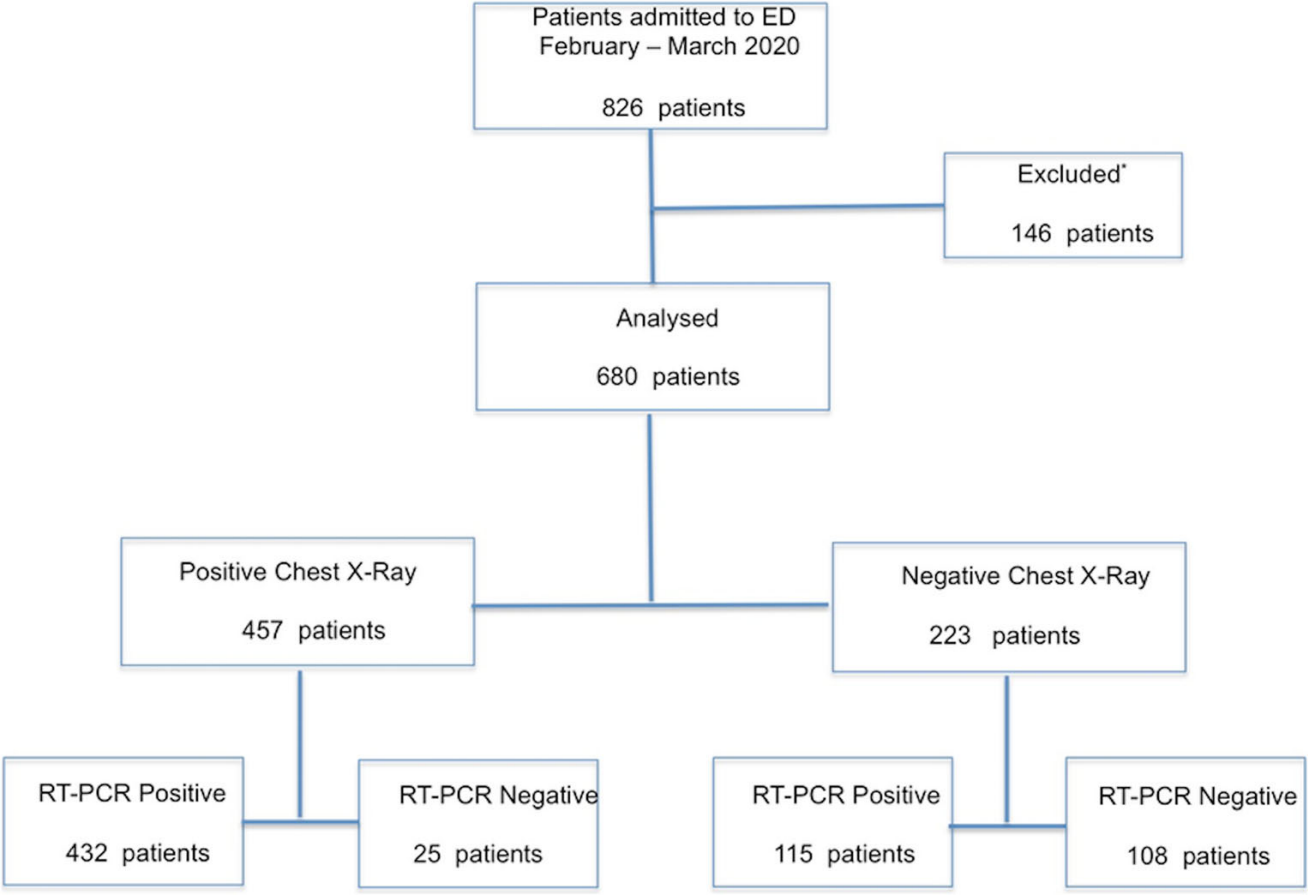
Patient flow chart. Out of the 826 consecutive patients who presented to the ED of our hospital between February 21 and March 31, 2020, 146 patients were excluded: 92 for not having clinical findings suspected of COVID-19 pneumonia, 50 because of the lack of rRT-PCR results and/or CXR, 4 because younger than 18

Out of the 826 consecutive patients who presented to the ED of our hospital between February 21 and March 31, 2020, 734 (89%) were clinically suspected of having COVID-19 and 680 were finally included in our analysis. A flow diagram is shown in Fig. 1.

Among these 680 patients, 528 were admitted to the hospital wards based on respiratory symptoms and laboratory test. Patients’ characteristics are described in Table 1.

**Table 1:** Characteristics of included patients. For sex, symptoms, and comorbidities, the number of patients (percentage) is reported in the right column. For age, the median age (interquartile range) is reported in the right column

		Number of patients	Median age
	All patients	680 (100%)	58 (IQR 44–71)
Sex	Males	412 (61%)	60 (IQR 47–70)
	Females	268 (39%)	55 (IQR 40–71)
Symptoms	Fever	552 (81%)	–
	Cough	380 (56%)	
	Dyspnoea	287 (42%)	
	Diarrhoea	56 (8%)	
	Asthenia	51 (8%)	
	Arthro-myalgias	36 (5%)	
	Pharyngodynia	37 (5%)	
	Low-grade fever	38 (6%)	
	Headache	34 (5%)	
	Nausea or vomiting	16 (2%)	
	Ageusia	12 (2%)	
	Anosmia	10 (2%)	
Comorbidities	All comorbidities	434 (64%)	–
	Hypertension	181 (27%)	
	Cardiovascular diseases among which: CAD	102 (15%)	
		49 (7%)	
	Diabetes mellitus	74 (11%)	
	Respiratory diseases	64 (9%)	
	Cancer	37 (5%)	
	Obesity	17 (3%)	

The patients who had a confirmation of COVID-19 based on rRT-PCR results were 547 (80%). In particular, among COVID-19 confirmed cases, 535 patients had a positive test at admission, while 12 patients had at least one first negative rRT- PCR before testing positive (eight patients had one, three had two, and one had three or more initial rRT-PCR negative tests), with an average delay of 3.2 days (range 1–8 days).

Among the 133 patients with negative rRT-PCR results, 55 patients had a single negative test, 31 had two consecutive negative tests, 34 had three, and 13 had more than three.

### Chest radiography diagnostic performance

CXR diagnostic performance for the expert reader and for the residents is summarized in Table 2.

**Table 2:** CXR diagnostic performance for the expert reader and for the radiology residents. Each value is presented as percentage [95% CI]. CXR was read by the expert reader as positive in 458 (67%) cases and as negative in 222 (33%) cases, with 432 true positive (TP) cases, 115 false negative (FN), 107 true negative (TN), and 26 false positive (FP). CXR was read by the non-expert readers as positive in 466 (69%) cases and as negative in 213 (31%) cases, with 410 TP, 136 FN, 77 TN, and 56 FP

	SE	SP	PPV	NPV	Accuracy	LR+	LR-
Expert reader	79% [75–82]	81% [73–87]	95% [92–96]	48% [44–53]	79% [76–82]	4,2 [2.9–6]	0.26 [0.22–0.31]
Residents	75% [71–79]	58% [49–67]	88% [86–90]	36% [32–41]	72% [68–75]	1,82 [1.48–2.24]	0.42 [0.35–0.52]

Comparing the reporting accuracy of the expert radiologist vs residents, sensitivity (79.0% vs 75.1%, *p* = 0.013) and specificity (80.5% vs 57.9%, *p* < 0.0001) were significantly different.

### Chest radiography diagnostic performance between subgroups

When we performed a subgroup analysis considering time from symptoms onset (> 5 days compared to ≤ 5 days, data available for 461 patients), we found that sensitivity was higher for patients with symptom onset > 5 days compared to ≤ 5 days (84.4% vs 70.7%, *p* = 0.002) while specificity was lower, although without statistical significance (72.9% vs 87.2%, *p* = 0.123). When we performed the analysis considering comorbidities (patients with comorbidities vs patients without comorbidities), we observed a significant difference in sensitivity (83.9% vs 74.3%; *p* = 0.006) but not in specificity (76.6% vs 82.6%; *p* = 0.494). We obtained similar results when considering the presence of cardiovascular comorbidities (sensitivity: 87.8% vs 77.4%, *p* = 0.039; specificity: 70.0% vs 81.3%, *p* = 0.41). No significant differences were observed when considering the presence of pulmonary comorbidities.

Finally, considering hospital admission, marker of severity of the disease, we observed a higher sensitivity and a lower specificity in admitted patients compared with not admitted patients (sensitivity: 82.6% vs 49.2%, *p* < 0.0001; specificity: 62.5% vs 88.2%, *p* = 0.001) (see Table 1 in Appendix).

### Diagnostic performance of chest radiography findings

The description of the diagnostic performance of specific CXR findings, namely, the twelve areas into which the lungs were divided and the three prevalent CXR patterns of lung abnormalities (interstitial reticular pattern, ground-glass opacities and extensive consolidations) is presented in Table 2 in Appendix.

From a multivariate logistic analysis including comorbidities, CXR patterns, and CXR areas, bilateral involvement of medium external lung areas emerged as independent predictor of the diagnosis of COVID-19 pneumonia (*p* = 0.032).

### Chest radiography findings and their distribution in positive patients

**Fig. 2:**
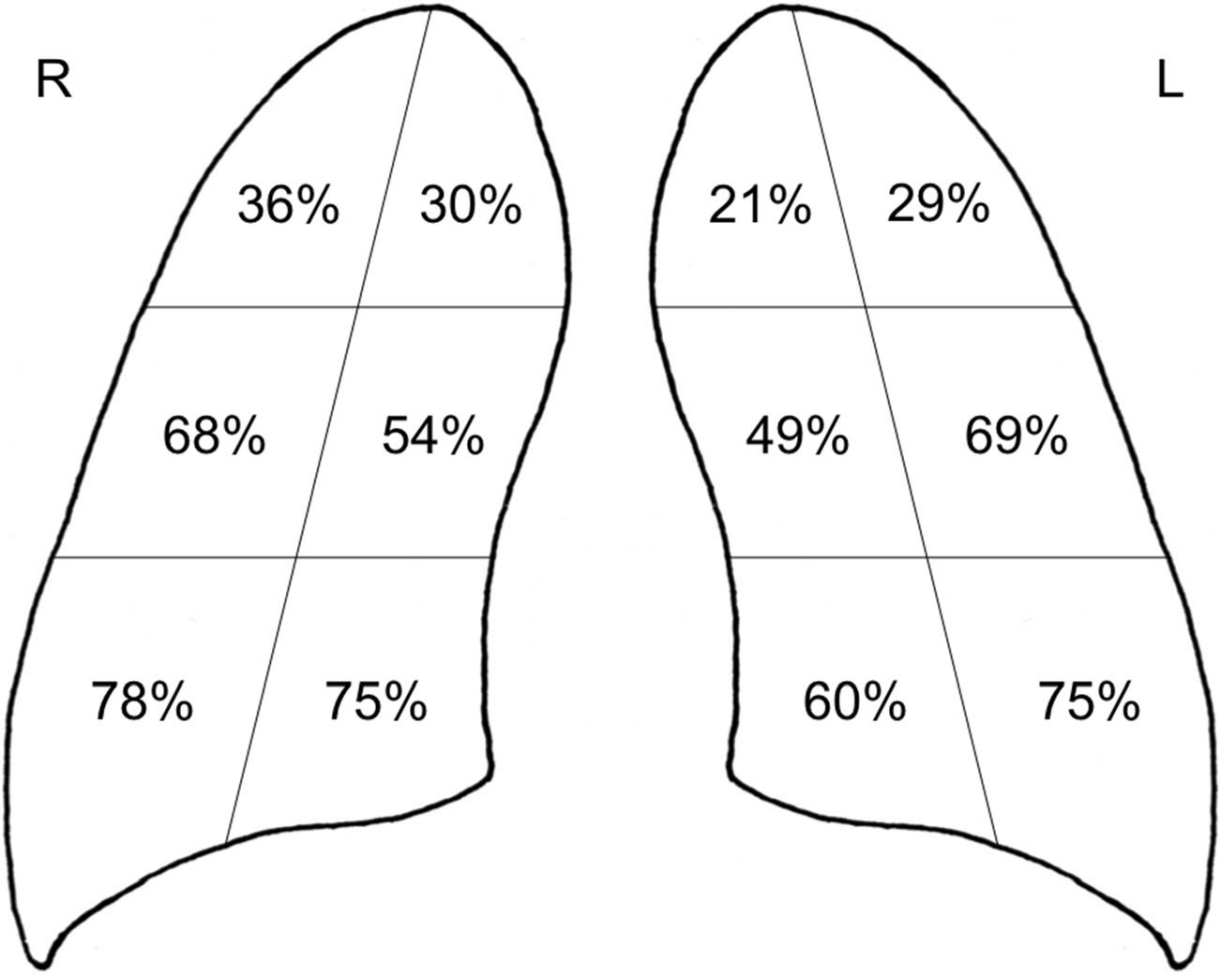
Distribution of typical findings at CXR. This figure describes the percentage of true positive patients with involvement of a given area, virtually dividing each lung into six areas. Considering the right lung (R), 156 (36% of 432) patients showed involvement of upper external portions, 130 (30%) of upper medial, 291 (68%) of middle external, 233 (54%) of middle internal, 335 (78%) of lower external, and 323 (75%) of lower internal portions. Considering the left lung (L), 126 (29%) patients showed involvement of upper external portions, 92 (21%) of upper medial, 297 (69%) of middle external, 209 (49%) of middle internal, 324 (75%) of lower external, and 260 (60%) of lower internal portion

**Fig. 3:**
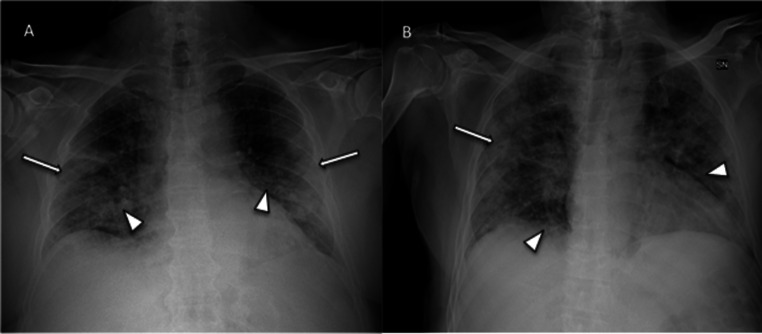
Chest radiography findings in two confirmed COVID-19 patients. CXRs in AP projection of two different patients (**A**, **B**) show subpleural ground-glass opacities (arrows) in external middle and lower lung areas and interstitial reticular pattern in internal areas (arrowheads). Both CXRs were judged as positive for COVID-19 by the expert reader and by the less expert readers; both patients had positive rRT-PCR results

**Fig. 4:**
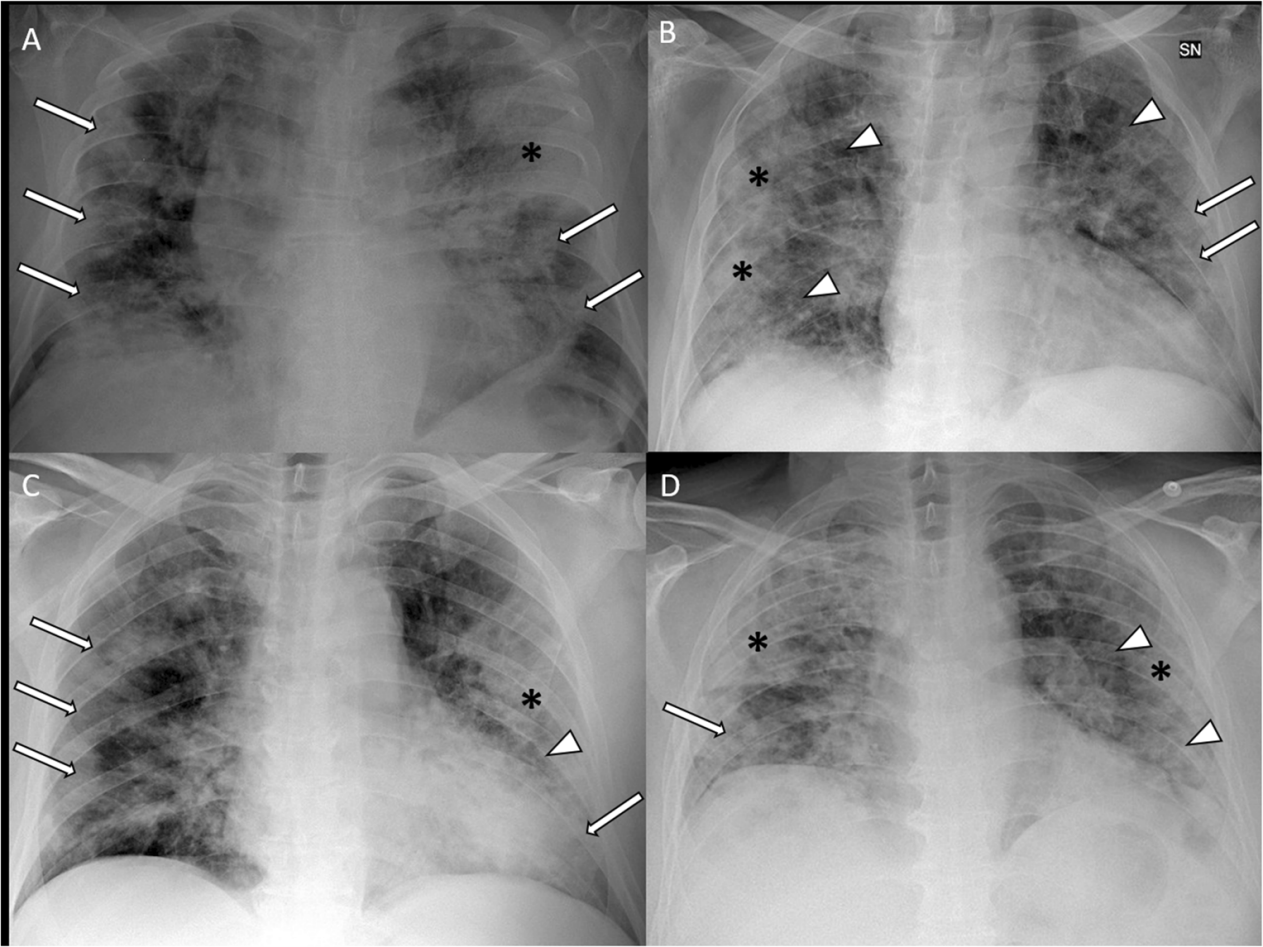
Chest radiography of four different confirmed COVID-19 patients, obtained at admission to the emergency department. CXRs in AP projection (**A**–**D**) show bilateral lung involvement with typical radiological patterns of COVID-19 pneumonia including the presence of ground-glass opacities (arrow), interstitial reticular alterations (arrowhead), and extensive consolidations (asterisk). All CXRs were interpreted as positive for COVID-19 by the expert reader and by residents. All four patients had positive rRT-PCR results

Among the COVID-19 confirmed patients, 79% had an abnormal CXR at the initial time of admission. The most common pattern was the interstitial reticular pattern (183 patients; 42%), followed by extensive consolidations (145 patients; 34%) and ground-glass opacities (104 patients; 24%). Men showed a significantly higher rate of extensive consolidations than women (37.5% vs 24.6%; *p* = 0.008). Extensive consolidations were also more frequently found in patients older than 60 (46% vs 20%; *p* < 0.0001). A detailed description of frequency and distribution of abnormalities in the 12 lung areas is shown in Fig. 2. CXR findings involved middle-lower lung portions in almost all patients (431; 99.5%), while involvement of upper portions was less common (201 patients; 46.5%). Figures 3 and 4 show examples of typical CXR findings with the typical lung distribution in confirmed COVID-19 patients. In 354 patients (82%), the involvement was bilateral. Among the 78 patients with unilateral distribution of disease, the right lung was more frequently involved than the left lung (68% vs 32%). Comparing patients with a positive CXR with patients with a negative CXR, we found a significant difference in age (*p* < 0.0001) and sex (*p* < 0.0001), positive patients being older and more frequently males.

### Discrepancies between CXR and rRT-PCR

One hundred fifteen patients had a positive rRT-PCR result without alterations on the initial CXR.

In 12 patients, positivity of CXR anticipated positivity of rRT-PCR by a mean of 3.2 days (range: 1–8 days).

Among the 25 false-positive patients (typical CXR abnormalities with negative rRT-PCR results), based on the combination of clinical and laboratory findings including serologic test (IgG) and chest CT, 7 were considered highly likely COVID-19 cases, 17 as probable cases, and one as a true false positive. When we considered the 7 highly likely cases as true positives, the CXR specificity increased up to 86%. Most of the false-positive patients had a bilateral involvement of middle-lower segments, with a predominance of reticular pattern.

## Discussion

Several centres worldwide are using CXR as the first-line imaging modality to evaluate symptomatic patients with clinical suspicion of COVID-19, but evidence on the performance of CXR in diagnosing COVID-19 pneumonia is limited. This consideration led us to describe our experience, in a setting characterized by a high disease prevalence, in a large patient cohort.

As the main result of our study, CXR interpreted by an expert radiologist showed a sensitivity of 79%, a specificity of 81%, and a PPV of 95% in diagnosing COVID-19 pneumonia, compared to rRT-PCR. Moreover, sensitivity of CXR was higher in patients who had had symptoms for more than 5 days compared with 5 days or less (84% vs 71%). These results suggest that, in a setting of high prevalence disease, a positive CXR in combination with clinical and laboratory findings can be sufficient at ED to triage symptomatic patients requiring hospital admission.

Comparing to alternative diagnostic algorithms that include chest CT instead of CXR, the choice of CXR has considerable advantages [4, 19]. Indeed, employing CT as a screening test entails a high contamination risk of the scanner during examination of infected patients, therefore increasing the risk of virus transmission to both hospital staff and other patients, who might later undergo examinations in the same CT scanner. Furthermore, the widespread adoption of CXR instead of CT to triage symptomatic patients would also have notable implications for countries with fewer resources and for centres with limited CT availability.

Our study was conducted in a high prevalence population, which could reduce the applicability of estimates to lower prevalence settings; dynamic changes of disease spectrum should also be considered when evaluating the imaging modality of choice for diagnosing COVID-19 pneumonia [20, 21]. Therefore, in other scenarios (i.e., low prevalence disease; future pandemic waves), chest CT could be useful in suspected patients with negative or uncertain CXR findings [18, 22].

As already mentioned, evidence on the performance of CXR in diagnosing COVID-19 is low. Wong et al. [11], by analysing less than 100 patients, reported, as the only measure, a CXR sensitivity of 69%; more recently, Ippolito et al. [12] obtained a mean accuracy slightly lower than ours; a similar result was reported by Schiaffino et al. [13], who used a composite reference standard which also included clinical parameters, instead of the conventional rRT-PCR alone. The differences that emerged in terms of sensitivity and specificity when we divided patients according to symptoms onset are similar to those reported by Ippolito et al. [12].

We found significant differences between the diagnostic performance of CXR interpreted by the expert radiologist and the diagnostic performance of CXR interpreted by the residents; in particular, the reader’s experience appeared to mainly influence specificity (81% vs 58%; *p* < 0.0001). This observation is consistent with similar previous investigations [23, 24] and can be explained by the fact that expert readers are more aware of other pathologies that can have a similar appearance on CXR, for instance, cardiogenic interstitial involvement or predominantly unilateral lung consolidations, expression of pneumonia of different aetiologies, and being able to correctly recognize them.

From our analysis of COVID-19 patients, as defined by a positive rRT-PCR result, emerged that patients with a positive CXR at admission were older and more frequently males, suggesting that these categories have a higher risk of pulmonary involvement by SARS-CoV-2. Among true positive patients, involvement of both lungs and of middle-inferior areas was seen in the vast majority of cases, a result consistent with previous studies [25]. Moreover, bilateral involvement of medium external lung areas was found to be an independent predictor of CXR diagnostic performance.

Our study has some limitations. Firstly, we should highlight that we described results of CXR interpretation performed by radiologists. Actually, many settings do not get reports of CXRs for days and CXRs are interpreted by the emergency healthcare workers, with an expected different diagnostic accuracy. Secondly, the gold standard diagnostic test we used as reference (rRT-PCR) is known to have a sensitivity ranging from 60 to 70%, and this constitutes a potential bias for diagnostic accuracy estimates. Thirdly, the retrospective nature of our study did not allow us to make a comparison with alternative diagnostic pathways. Finally, we did not investigate the prognostic value of CXR for clinical outcomes such as mortality, hospitalization length, or risk of intubation; this investigation is the main objective of a further article.

In conclusion, in this study, chest radiography demonstrated a sensitivity of 79%, a specificity of 81%, and a PPV of 95% in diagnosing viral pneumonia in symptomatic patients with clinical suspicion of COVID-19, in a setting of high disease prevalence. To verify the applicability of our results, further similar studies conducted preferably in settings of lower disease prevalence are needed.

## Supplementary Information

ESM 1 (DOCX 13 kb)

ESM 2 (PNG 4653 kb)

High resolution image (TIFF 53053 kb)

ESM 3 (PNG 2768 kb)

High resolution image (TIFF 35794 kb)

## Data Availability

Dataset from this study is available from the corresponding author.

## Abbreviations

rRT-PCR: Real-time reverse transcription-polymerase chain reaction
ED: Emergency department
SARS- CoV-2: Severe acute respiratory syndrome coronavirus 2
COVID-19: Coronavirus disease 2019
CXR: Chest radiography
LUS: Lung ultrasound
