# Supplementary material for: Diagnostic performance of chest radiography in high COVID-19 prevalence setting: experience from a European reference hospital
Source: Emerg Radiol. 2021 Jul 3;28(5):877–85. doi: 10.1007/s10140-021-01946-x (PMC8254671; doi:10.1007/s10140-021-01946-x)
Supplement: Supplementary file 1 — (DOCX 13 kb) [file 10140_2021_1946_MOESM1_ESM.docx]

**Appendix tables, legends**

**Table 1.** This table describes, for the group of patients admitted to hospital wards and for those discharged from ED, the diagnostic performance of: expert radiologist, the CXR patterns of lung abnormalities (interstitial reticular pattern, ground-glass opacities and extensive consolidations) and the twelve lung areas.

**Table 2.** This table describes the diagnostic performance of: expert radiologist, the twelve lung areas and the CXR patterns of lung abnormalities (interstitial reticular pattern, ground-glass opacities and extensive consolidations).
