# Supplementary figures and images for: Diagnostic performance of chest radiography in high COVID-19 prevalence setting: experience from a European reference hospital
Source: Emerg Radiol. 2021 Jul 3;28(5):877–85. doi: 10.1007/s10140-021-01946-x (PMC8254671; doi:10.1007/s10140-021-01946-x)

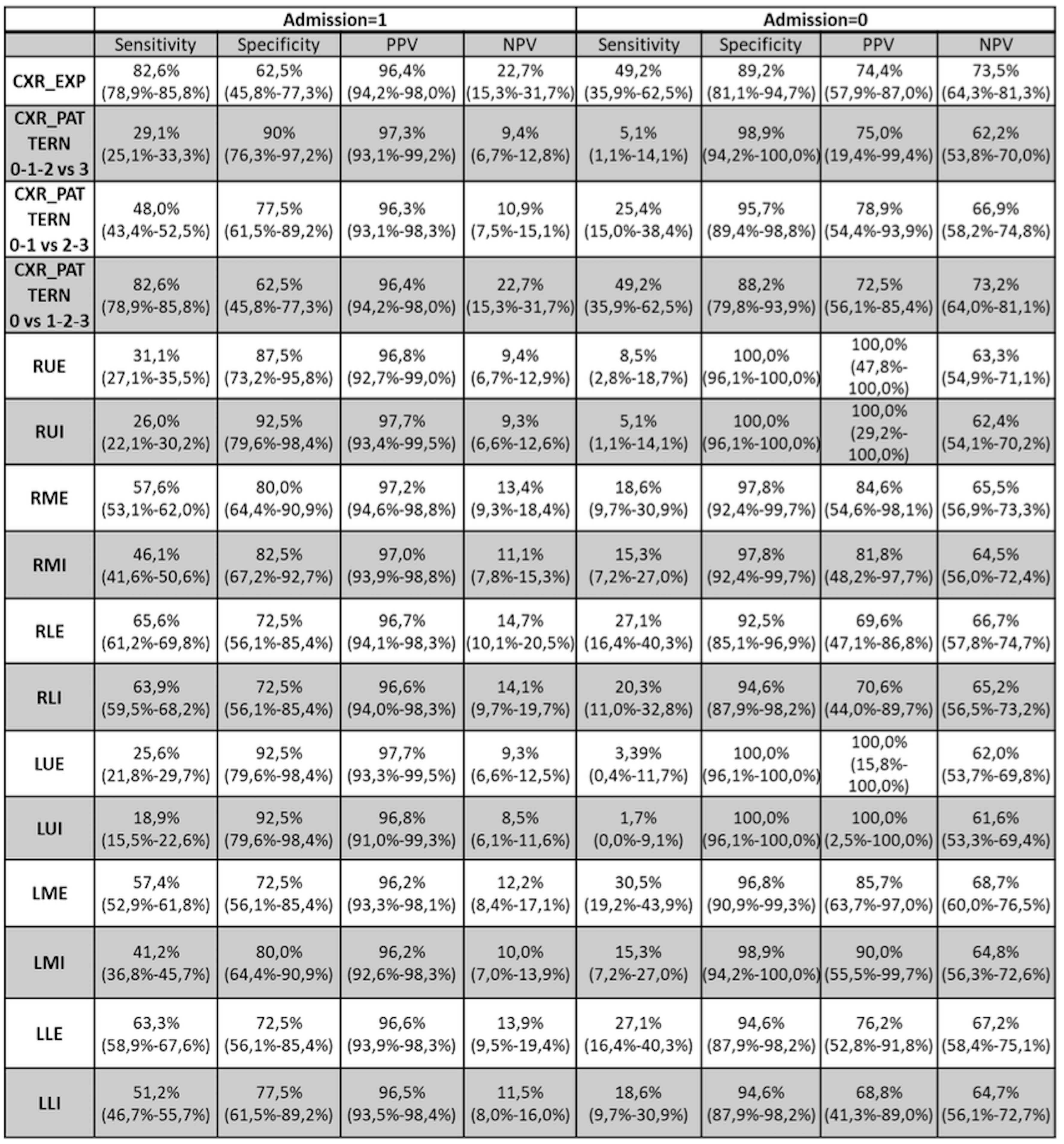

Supplement: Supplementary file 2 — (PNG 4653 kb) [file 10140_2021_1946_Fig5_ESM.png]

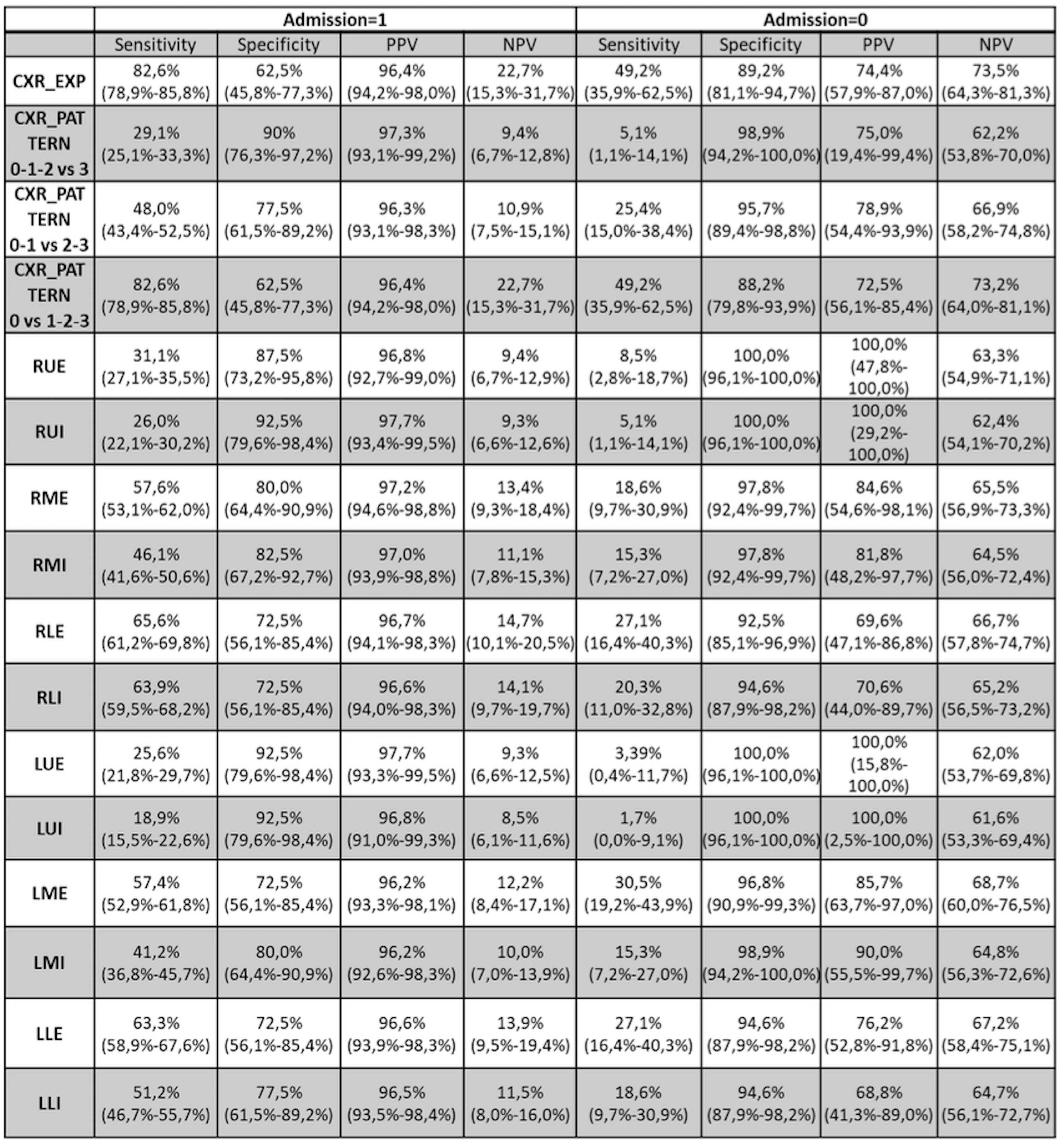

Supplement: Supplementary file 3 — High resolution image (TIFF 53053 kb) [file 10140_2021_1946_MOESM2_ESM.tiff]

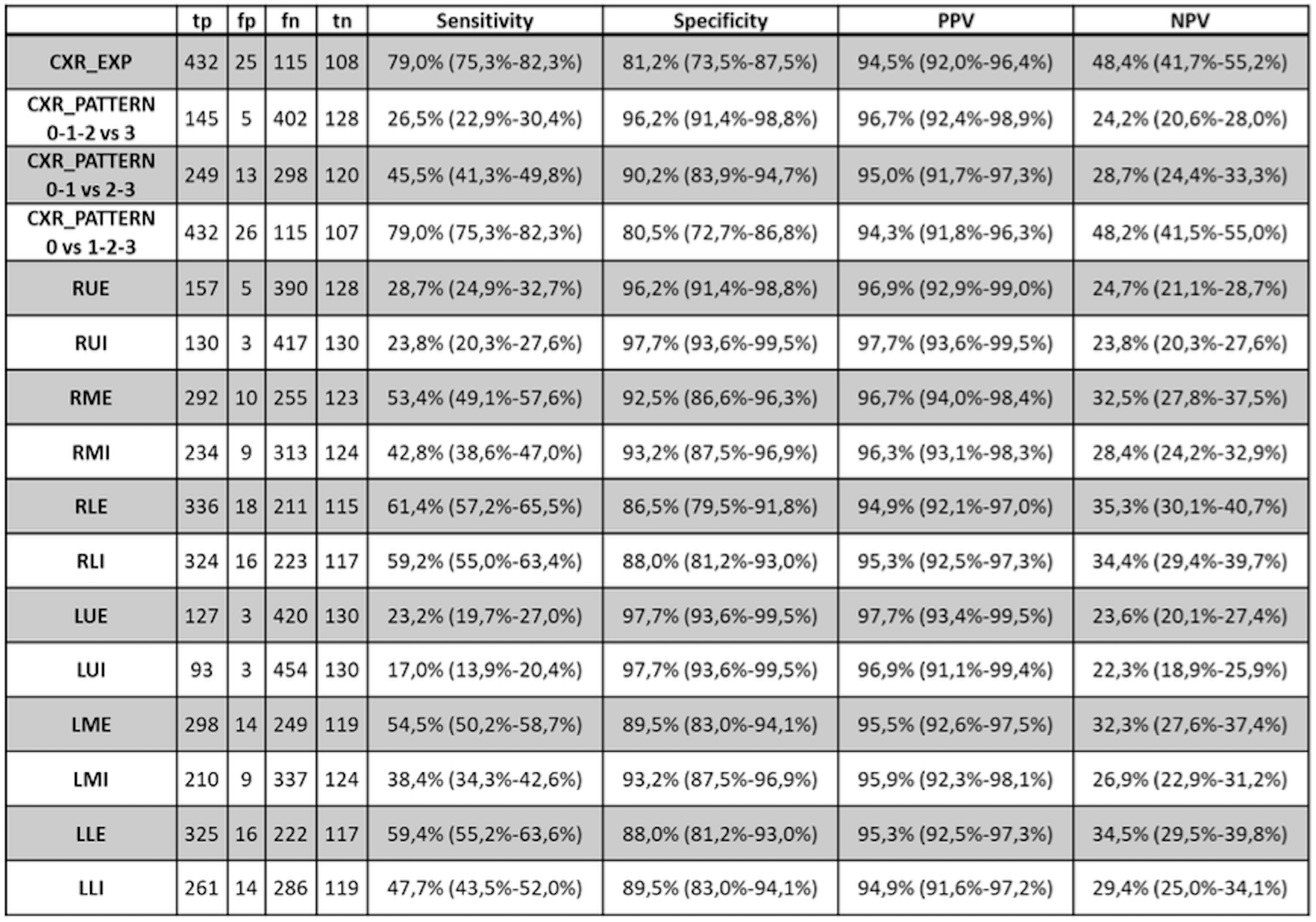

Supplement: Supplementary file 4 — (PNG 2768 kb) [file 10140_2021_1946_Fig6_ESM.png]

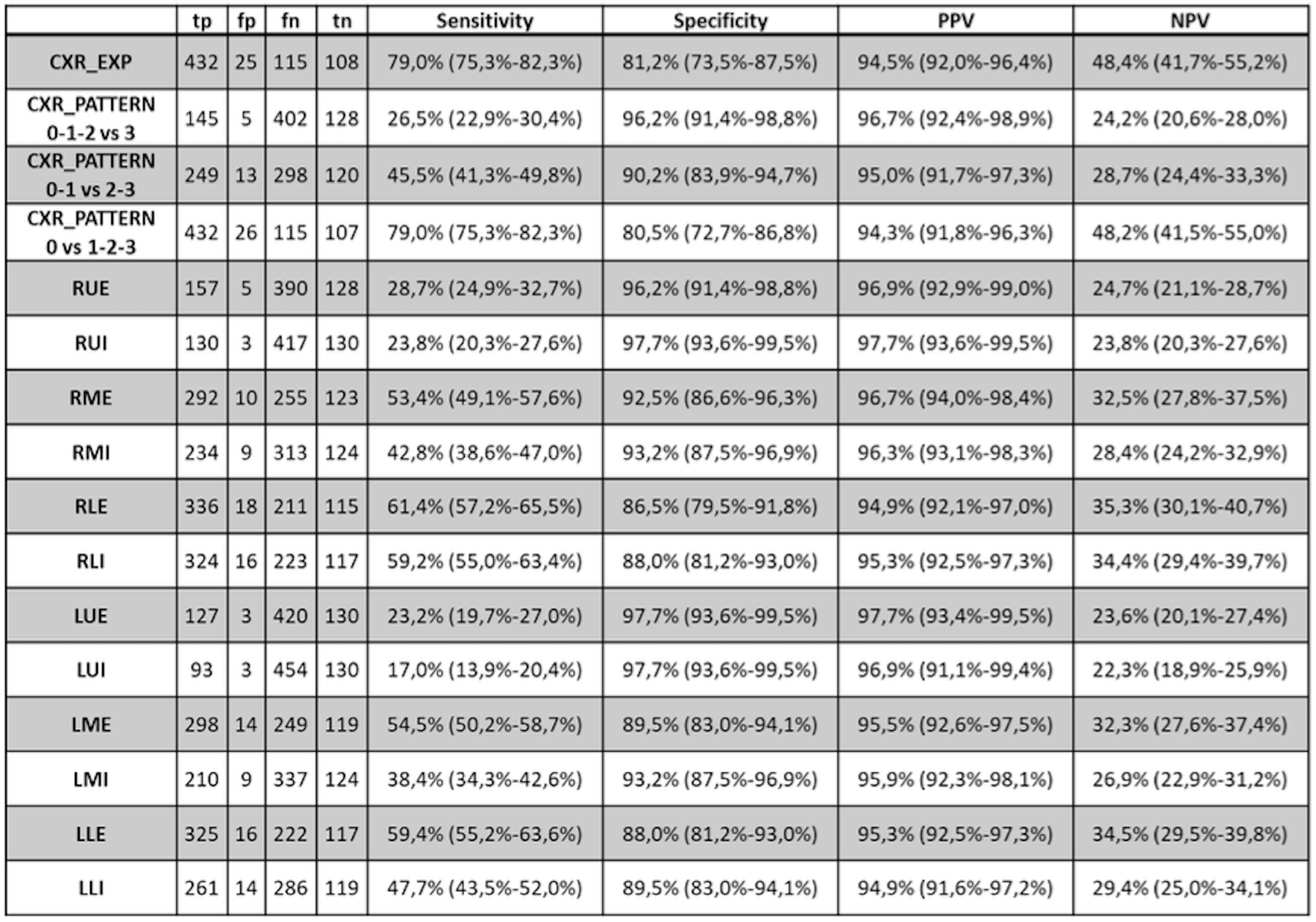

Supplement: Supplementary file 5 — High resolution image (TIFF 35794 kb) [file 10140_2021_1946_MOESM3_ESM.tiff]
